# Risk Factors for Short Stature in Children Born Small for Gestational Age at Full-Term

**DOI:** 10.3389/fped.2022.833606

**Published:** 2022-06-22

**Authors:** Lan Ling, Ting Chen, Xin-Hua Zhang, Min-Hong Pan, Hai-Hong Gong, Li-Na Zhang, Meng Zhao, Xiao-Qing Chen, Shu-Dong Cui, Chao Lu

**Affiliations:** ^1^Department of Pediatrics, The First Affiliated Hospital of Nanjing Medical University, Nanjing, China; ^2^Department of Radiology, The First Affiliated Hospital of Nanjing Medical University, Nanjing, China; ^3^Department of Children's Health Care, The First Affiliated Hospital of Nanjing Medical University, Nanjing, China; ^4^Department of Pathology, The First Affiliated Hospital of Nanjing Medical University, Nanjing, China

**Keywords:** short, small for gestational age, full-term, children, risk factor

## Abstract

**Objective:**

This study aims to identify the risk factors associated with short stature in children born small for gestational age (SGA) at full-term.

**Methods:**

This was a retrospective study. The subjects were full-term SGA infants who were followed up until the age of 2 years. The risk factors for short stature were identified with univariate and multivariate analyses.

**Results:**

Of 456 full-term SGA children enrolled in this study, 28 cases had short stature at 2 years of age. A significant decrease in placental perfusion was found in the short children group with intravoxel incoherent motion (IVIM) technology, which was an advanced bi-exponential diffusion-weighted imaging (DWI) model of magnetic resonance imaging (MRI) (*p* = 0.012). Compared to non-short children born SGA at full-term, the short children group underwent an incomplete catch-up growth. Mothers who suffered from systemic lupus erythematosus were more likely to have a short child born SGA (*p* = 0.023). The morbidity of giant placental chorioangioma was higher in the short children group. The pulsatility index (PI), resistivity index (RI), and systolic-diastolic (S/D) ratio of umbilical artery were higher in the short children group than in the non-short control group (*p* = 0.042, 0.041, and 0.043). Multivariate analysis demonstrated that decrease of perfusion fraction (*f*_p_) in IVIM of placental MRI, chromosomal abnormalities, short parental height, and absence of catch-up growth were associated with a higher risk of short stature in children born SGA at full-term.

**Conclusion:**

Risk factors for short stature in full-term SGA children at 2 years of age included a decrease of perfusion fraction *f*_p_ in IVIM of placental MRI, chromosomal abnormalities, and short parental height.

## Introduction

Small for gestational age (SGA) is defined as birthweight and length <10th percentile for gestational age (1, 2). According to different gestational age, SGA infants are divided into full-term, preterm, and post-term. Full-term SGA infants comprise a heterogeneous group with a broad spectrum of clinical characteristics and are at increased risk of perinatal morbidity, persistent short stature, and metabolic alterations in later life. However, approximately 90% of SGA infants show catch-up growth during the first years of life and achieve sufficient growth to normalize their stature by 2 years of age. The remaining 10% have a height below−2 SD score throughout adolescence and have short stature in adulthood (3, 4). Therefore, it is essential to identify any risk parameters for predicting the outcomes of full-term SGA children at 2 years of age and to determine the optimal time for initiating growth hormone therapy.

Some clinical research has demonstrated that reduced size at birth may result from fetal, maternal, and/or placental factors (5–7). However, it is still currently difficult during the perinatal period, to predict precisely which child will have short stature at 2 years of age. Functional magnetic resonance imaging (MRI) methods may help to diagnose placental insufficiency. Indeed, an advanced bi-exponential diffusion-weighted imaging (DWI) model, the intravoxel incoherent motion (IVIM) MRI, can be used to quantify placental perfusion and diffusion (8, 9). For evaluating placental perfusion, IVIM employs some functional parameters, including perfusion fraction *f* and *D*, which represents capillary perfusion and vessel flow velocity respectively (8). Recently, our study validated that the perfusion fraction *f*_p_ can be utilized to distinguish fetal growth-restricted pregnancies from normal pregnancies (9). IVIM was helpful to improve the diagnosis of placental dysfunction.

A 6.8–11.4% increase of chromosomal abnormalities at karyotyping was demonstrated in SGA patients at full-term compared to normal infants (7, 10, 11). Ruan Peng *et al*. reported the chromosomal and sub-chromosomal anomalies associated to SGA fetuses (10). SGA infants experience fetal growth restrictions, especially those born full-term (7, 10). The long-term influence of chromosomal abnormalities on growth and height of full-term SGA children after birth deserves further investigation.

The aim of the present study was to investigate the risk factors for short stature in full-term SGA children. Identified factors that cause growth impairment in these children may enable us to find therapeutic or preventive options.

## Materials and Methods

This retrospective study was performed at Jiangsu province hospital in China. The children born between 1 January 2016 and 30 November 2019, who were followed up to 2 years of age and met the following criteria, were included. The inclusion criteria were: 1. Gestational age ≥ 37 weeks and < 42 weeks; 2. SGA, defined as birthweight < 10th percentile for gender and gestational age by Chinese birthweight growth charts; 3. clinical follow-up available until 2 years of age (4). The exclusion criteria included congenital malformation and genetic metabolic disease. Renal tubular acidosis, vitamin D-resistant rickets, and other metabolic diseases which may cause short stature were excluded. Endocrine diseases, such as thyroid and adrenal disorders, were also excluded. Standard deviation (SD) and Z scores of heights at 12, 18, and 24 months of age were calculated according to previous studies (12). ΔZ score was defined as the change in the Z score for length or height during the last year. Catch-up growth was defined as ΔZ score > 0.67 SD or height above−2SD or height above 3 percentiles during the first two years of life. A child was considered of short stature if the height was below−2 SD for gender at 2 years of age. Mid-parental height was calculated as described previously by Lu (13).

As we described previously, all pregnant women at 20–34 weeks of gestation were examined with a 1.5T magnetic resonance (MR) scanner (9). MR protocols included a T1-weighted gradient echo sequence in sagittal direction, T2-weighted turbo spin echo sequence in coronal, transversal, and sagittal directions, and T2-weighted balanced steady state free procession sequence. IVIM of MRI was performed based on a single-shot echo-planar imaging sequence [repetition time (TR)/echo time (TE) = 4000/73.6 msec; echo spacing, 0.58 msec; slice thickness, 4 mm; in-plane resolution, 3.5 × 3.5 mm^2^; total acquisition time, 5.5 min] with a spectrum of different beta-values (b-values) of 0, 20, 40, 80, 160, 200, and 500 s/mm^2^. The image analysis of IVIM post-processing based on the multi b-value diffusion weighted images were performed using Osirix software (Osirix, https://www.osirix-viewer.com/osirix/osirix-md/) with the following bi-exponential model (9). An equation was utilized as follows:


$$\begin{array}{l} S\left(b\right)=S0\cdot \left[\left(1-{\text{f}}_{\text{p}}\right)\cdot exp\left(-b\cdot {\text{D}}_{\text{t}}\right)+{\text{f}}_{\text{p}}\cdot exp\left(-b\cdot {\text{D}}_{\text{p}}\right)\right], \end{array}$$

where S(b) is the signal intensity at a given b-value and S0 is the signal intensity at b-value = 0 s/mm^2^. As a perfusion fraction, *f*_p_ stands for the volume fraction of the vascular compartment with its corresponding diffusion coefficient, Dp (pseudodiffusivity). (1- *f*_p_) stands for the remaining volume fraction, tissue or cellular compartment, with its corresponding diffusion coefficient, *D*_t_ (true diffusivity) (9).

The ultrasound examination at 32 ± 2 weeks of gestation was performed by trained sonographers and included fetal biometry and Doppler studies of the umbilical and uterine arteries. Pulsatility index (PI), resistivity index (RI), and the systolic-diastolic ratio (S/D) were recorded. The changes of PI, RI, and *fp* of IVIM was evaluated with the cut-off values from receiver operating characteristic (ROC) curves and area under the curve (AUC) analysis as previous described (9, 14).

Chromosome karyotyping and DNA sequencing were performed as previously described with peripheral blood (7, 10, 11). The placentas were examined routinely and the sizes of tumors were measured after delivery. All specimens were fixed in 4% formaldehyde for 48 h and were then embedded in paraffin, sectioned, and routine Hematoxylin-Eosin (HE) staining was performed.

### Statistical Analysis

All statistical analyses were performed using SPSS (Statistical Package for Social Sciences, version 23) software. Quantitative variables were expressed as means ± SD. Qualitative variables were expressed as percentages. Student's *t*-test was used for analysis of normally distributed variables, while the Mann-Whitney U-test was used for analysis of non-normally distributed variables. The differences between groups for proportions were assessed by the χ^2^ test. The Fisher exact test was used as a replacement for the chi-square test when the expected frequency of one or more cells was <5. Risk factors with *p* < 0.01 in univariate analysis were available for inclusion in the multivariate model. Multivariate analysis was performed using stepwise logistic regression. A *p* value < 0.05 was considered statistically significant.

## Results

A total of 1,646 children were SGA, representing 5.25% of all children born alive in Jiangsu province hospital during the study period. Of these, there were 692 full-term SGA infants and 954 preterm SGA infants. According to the inclusion and exclusion criteria, 456 full-term SGA children were enrolled in this study, including 428 children with normal height and 28 children with a short stature at 2 years of age. The clinical features of the 28 short children are shown in Table 1. Among full-term SGA children, there were no significant differences in gravidity, parity, gestational age, and birth length between male and female infants.

**Table 1:** The clinical features of 28 full-term small for gestational age (SGA) children with short stature enrolled in this study.

	**Male (*n* = 15)**	**Female (*n* = 13)**	***p*-value**
Parity (*n*)	1.2 ± 0.2	1.2 ± 0.3	0.895
Pregnancy (*n*)	1.6 ± 0.8	1.6 ± 0.9	0.844
Gestation age (weeks)	38.3 ± 1.4	38.6 ± 1.1	0.786
Birthweight (g)	2299.6 ± 321.2	2328.3 ± 295.5	0.048
Birth length (cm)	45.8 ± 1.9	46.4 ± 1.6	0.056
Weight at age 2 (kg)	10.52 ± 1.21	9.88 ± 1.18	0.042
Height at age 2 (cm)	79.3 ± 1.5	78.4 ± 1.6	0.049
Height at age 2 Z score	−3.1 ± 0.8	−3.1 ± 0.9	0.684

*Data are represented as mean ± standard deviation*.

A univariate analysis was performed to identify the potential risk factors for short stature in children born SGA, and the results are shown in Table 2. As expected, the ΔZ score for height at 12 and 18 months of age (between 0 and 12 months, 0 and 18 months) was significantly lower in short children born SGA at full-term compared to non-short control (*p* = 0.018; *p* = 0.016, respectively). The maternal and paternal heights were significantly lower in short children born SGA at full-term compared to non-short control (*p* = 0.011, 0.021). There were no significant differences in birth weight and length between the two groups (*p* = 0.112, 0.699). Similar results were observed in Z scores of birth weight and length between the groups (*p* = 0.521, 0.589).

**Table 2:** The univariate analysis of risk factors for short stature in children born SGA at full-term.

	**Short children born SGA (*n* = 28)**	**Non-short children born SGA (*n* = 428)**	***p*-value**
**Birth information**			
gestation age (weeks)	38.6 ± 1.3	38.4 ± 1.4	0.794
Sex (Male/Female)	15/13	222/206	0.875
Apgar score at 1 min	8.3 ± 1.5	8.4 ± 1.4	0.839
Birthweight (g)	2312.4 ± 308.1	2358.3 ± 312.6	0.112
Birth length (cm)	46.1 ± 1.8	46.9 ± 1.5	0.699
Father's height (cm)	162.9 ± 2.6	170.9 ± 5.2	0.011
Mother's height (cm)	153.4 ± 3.4	159.7 ± 4.3	0.021
**Chromosome abnormality**	6 (21.4%)	3 (0.7%)	0.000
**Neonatal diseases and complications**			
Asphyxial	4 (14.3%)	15(3.5%)	0.023
Hypoglycemia	5 (17.9%)	38 (8.9%)	0.169
Anemia	6 (21.4%)	45 (10.5%)	0.111
**Maternal information**			
Chronic hypertension	2 (7.1%)	8 (1.7%)	0.121
Systemic lupus erythematosus	4 (14.3%)	15 (3.5%)	0.023
**Pregnancy information**			
Gestational diabetes mellitus	5 (17.9%)	58 (13.6%)	0.569
Chorioamnionitis	2 (7.1%)	21 (4.9%)	0.644
Giant placental chorioangioma	2 (7.1%)	2 (0.5%)	0.021
**Ultrasound parameter for umbilical artery**			
Pulsatility index	1.39 ± 0.31	1.28 ± 0.22	0.042
Resistance index	0.88 ± 0.15	0.75 ± 0.13	0.041
Systolic-diastolic ratio (S/D)	3.96 ± 1.37	3.74 ± 1.21	0.043
**Catch-up growth rates**			
12m length ΔZ score	−1.6 ± 0.6	0.8 ± 0.5	0.018
18m length ΔZ score	−1.0 ± 0.2	0.9 ± 0.6	0.016
**IVIM parameter for placental perfusion**			
*f*_p_ (%_)_	18.24 ± 7.56	23.11 ± 9.43	0.012
*D*_t_ (10^3^mm^2^/s)	1.11 ± 0.18	1.36 ± 0.23	0.043
*D*_p_ (10^3^mm^2^/s)	44.09 ± 40.33	49.78 ± 43.69	0.041

*Data are represented as mean ± Standard Deviation or Number of cases (%). IVIM, Intravoxel Incoherent Motion; f_p_, Perfusion fraction; D_t_, True diffusivity; D_p_, Pseudo diffusivity*.

The data of Doppler indices from the scans at 32 ± 2 weeks of gestation demonstrated that umbilical artery PI, RI, and S/D ratio were higher in the short children group than those in the non-short children born SGA group as a control (*p* = 0.042, 0.041, and 0.043). This result indicated that the short children in the fetal stage underwent a significant decrease of diastolic flow in the umbilical artery, which was due to an increase in the resistance that might occur in small arteries and arterioles of the placenta.

**Figure 1:**
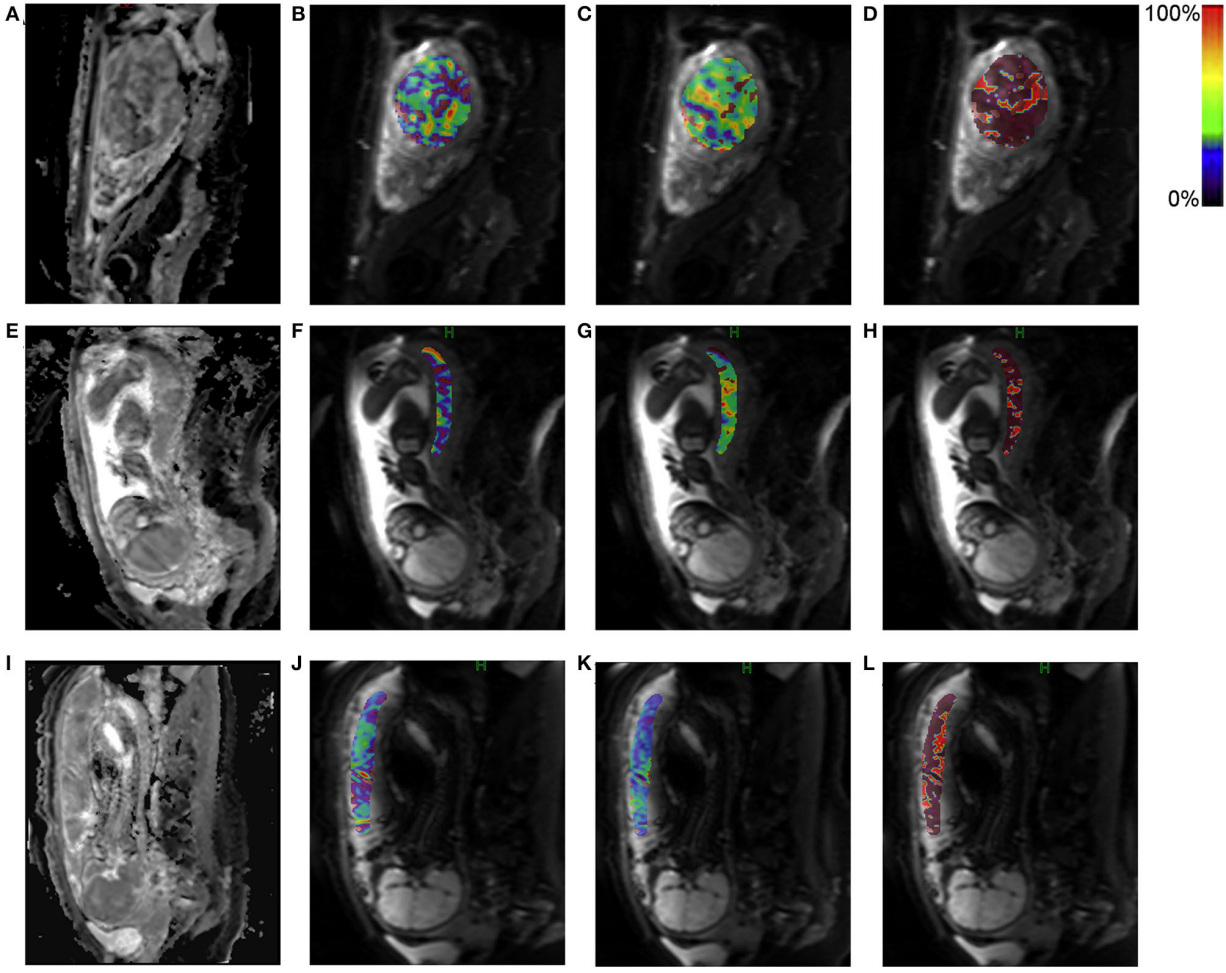
Representative intravoxel incoherent motion (IVIM) parametric maps for placental perfusion. The upper panel shows the IVIM parametric maps of a pregnant woman with a normal placental perfusion. The middle and lower panels respectively show low and lower placental perfusion of different pregnant women. **(A,E,I)**: Diffusion weighted image at b-value = 0 mm^2^/s; **(B,F,J)**: Perfusion fraction *f*_*p*_; **(C,G,K)**: True diffusivity *D*_*t*_; **(D,H,L)**: Pseudo diffusivity *D*_*p*_. The region of interest was drawn on diffusion weighted image (b = 0 mm^2^/s) and then transferred to the other maps with corresponding pseudo color bar indicating the range of value.

In the short children group, the perfusion fraction *f*_p_ of IVIM parameters was 18.24 ± 7.56 %, which was significantly lower than that in non-short children group, 23.11 ± 9.43% (*p* = 0.012). Moreover, similar difference was also observed for the *D*_t_ and *D*_p_ (*p* = 0.043, 0.041) between the two groups. The decreased values of *f*_p_, *D*_t_, and *D*_p_ signified a hypo-perfusion and a decreased capillary flow velocity in the placenta. Representative IVIM parametric maps, including *f*_p_, *D*_t_, and *D*_p_, are displayed in Figure 1.

A cut-off value for short children born SGA was identified by ROC curve and AUC with mid-parental heights. The result of analysis indicated that the cut-off value was 157.1 cm. The sensitivity and specificity were 91.2 and 90.1%, respectively. Chi-squared tests demonstrated that mid-parental height was not related to the changes of PI, RI, or IVIM (Table 3). However, short mid-parental height was associated with the low ΔZ scores of height at 12m, 18m and 24m (Table 4), suggesting the low catch-up growth rate seen in SGA children was associated short parents.

**Table 3:** The chi squared test of mid-parental height against pulsatility index (PI), resistivity index (RI), or intravoxel incoherent motion (IVIM) measures.

	**Mid-parental height in short children born SGA (*****n*** **=** **28)**		***X* ^2^**	***p*-value**
	**≤157.1 cm (*n* = 8)**	**> 157.1 cm (*n* = 20)**		
PI
Increase	3	3
Non-increase	5	17	1.718	0.311
RI
Increase	3	4
Non-increase	5	16	0.933	0.371
*f*_p_ of IVIM
Decrease	4	5
Non-decrease	4	15	1.637	0.372

*PI, Pulsatility index; RI, Resistance index; IVIM, Intravoxel Incoherent Motion; f p, Perfusion fraction*.

**Table 4:** The chi squared test of catch-up growth vs. the classification of parental short stature.

**Catch-up growth rates**	**Mid-parental height in the total study population (*****n*** **=** **456)**		***X* ^2^**	***p*-value**
	**≤157.1 cm (*n* = 14)**	**> 157.1 cm (*n* = 442)**		
12 m length ΔZ score
≤ 0.67 SD	11	29
> 0.67 SD	3	413	87.933	0.000
18 m length ΔZ score
≤ 0.67 SD	10	24
> 0.67 SD	4	418	85.663	0.000
24 m height ΔZ score
≤ 0.67 SD	8	20
> 0.67 SD	6	422	65.190	0.000

Karyotypic analysis and whole exon sequencing showed that the proportion of cases with chromosomal abnormalities or gene mutations was 21.4% (6/28) in short SGA children. It was 0.7% in the catch-up growth group (*p* = 0.000). The chromosomal abnormalities included 47, XXX; 47, XY, +8(15)/46; 45, XO (5)/46; deletion of 15q11-q13 and other abnormal karyotypes. With methylation-specific polymerase chain reaction (PCR) technique, a case with hypomethylation of the imprinted control region 1 (ICR1) at chromosome 11p15.5 was observed. Compared to control (0.7%), this higher frequency indicated a strong association between chromosome aberration and short stature in full-term SGA (*p* < 0.001).

Compared with non-short SGA children, short children had a higher incidence of asphyxia in the perinatal period (14.3%, *p* = 0.023), while the morbidities did not differ in intrauterine infection, hypoglycemia, anemia, and intraventricular hemorrhage during the neonatal period between the two groups.

Mothers who suffered from systemic lupus erythematosus were more likely to have a short child born SGA (*p* = 0.023). There were no significant differences in the occurrence rates of chronic hypertension and anemia in mothers of both study groups. No significant difference was found in the prevalence of preeclampsia, gestational diabetes mellitus, and chorioamnionitis.

There were two cases with giant placental chorioangioma in the group of short children born SGA at full-term. The sizes of the masses were 12 × 9 × 7.5 cm and 11 × 7 × 4 cm. This result suggested that giant placental chorioangioma might be associated with short stature in children born SGA.

Multivariate logistic regression analysis was next performed with the significant factors on univariate analysis, and the result is shown in Table 5. In a final model, four variables were associated with an increased risk of short stature, and the other two were protective. Short parental height was a significant risk factor for short stature in the children with full-term SGA. Excluding the ΔZ scores, short parental height, decrease of IVIM *f*_p_, and chromosome abnormality were still related to the risk.

**Table 5:** Multivariate analysis of risk factors for short stature in children born SGA at full-term.

**Risk factors**	**OR**	**95% CI**	***p*–value**
Father's height (cm)	2.122	1.842–3.517	0.025
Mother's height (cm)	2.564	1.243–3.982	0.024
IVIM *f*_p_ (%)	1.668	1.224–2.486	0.042
12 m length ΔZ score	0.325	0.186–0.512	0.001
18 m length ΔZ score	0.104	0.026–0.219	<0.001
Chromosome abnormality	5.546	1.288–9.752	<0.001

*Data are represented as odds ratio (OR) for developing short stature with 95% confidence intervals. P values <0.05 are considered significant. IVIM, Intravoxel Incoherent Motion; f_p_, Perfusion fraction*.

Multivariable analysis demonstrated that the decrease of perfusion fraction *f*_p_ in IVIM parameters resulted in a significant odds ratio (OR) of 1.668 for development of short stature (*p* = 0.042), indicating that placental insufficiency was an important risk factor. Furthermore, we found a 5.5-times increased probability of short stature at the age of 2 years when full-term SGA infants had a chromosomal abnormality [OR: 5.546, 95% confidence interval (CI): 1.288–9.752, *p* < 0.001].

## Discussion

Ultrasonography is the first-line imaging modality for determining placental insufficiency and fetal growth retardation by measuring PI, RI, and S/D (15). In the present study, our results showed that fetal umbilical artery PI, RI, and S/D ratio were higher in the short children group compared to those in the non-short children born SGA group. However, ultrasound examination is also an experience-dependent diagnostic technique, of which sensitivity and repeatability remain to be improved for determining placental perfusion. IVIM is an advanced bi-exponential DWI model of MRI, which holds great potential in evaluating placental insufficiency, especially in quantifying perfusion and diffusion (8, 9). In this study, both univariate and multivariate analyses demonstrated that a decreased perfusion fraction *f*_p_ of IVIM parameters was significantly associated with the risk of short stature in the children born SGA at full-term. We reported previously that there existed a negative correlation between *f*_p_ and umbilical artery PI (9). Together with these data, our finding suggests that combined utilization of the two methodologies could provide complementary information on placental circulation and benefit prediction of risk for short stature in SGA children. It should be noted that the two cases that suffered from giant placental chorioangioma in this study also had an increased umbilical artery PI, which was in accordance with previous literature (16). Giant placental chorioangioma tumors cause physiological and functional dead space and result in the decrease of normal placenta size (16). This may lead to uteroplacental insufficiency with subsequent fetal growth restriction and deliveries of SGA infants. D. Buca et al. reported that the diagnosis of SGA fetus on prenatal ultrasound was made in 21.4% of pregnancies complicated by placental chorioangioma (17). However, the association between giant placental chorioangioma and short stature in SGA children requires further investigation with larger samples.

In the present study, our results demonstrated that both parental height and chromosomal abnormality were associated with short stature in the SGA children. There was a 5.5-fold increased risk in the babies with chromosomal abnormality. This finding was in line with previous studies that fetal growth impairment is associated with a chromosomal aberration detected by karyotyping and microarray in 2–19% of cases (18). Along with the development of high-throughput DNA-scanning techniques, genetic causes have been frequently found in short SGA children, such as point mutations in the insulin-like growth factor 1 (IGF1) (19). Short stature is one of the most important clinical manifestations of Prader-Willi syndrome, which is a multisystemic complex genetic disorder caused by lack of expression of genes on the paternally inherited chromosome 15q11.2-q13 region (20, 21).

In this study, we found that mid-parental height was not related to the changes of PI, RI, or IVIM. This result implied that short mother did not necessarily have a utero-placental insufficiency, but short mid-parental height was associated with the decreases of 12 m, 18 m length ΔZ score and 24 m height ΔZ score, indicating that catch-up growth rates were low in SGA children with short parents. Although mid-parental height is a surrogate of genetic effects and does not reflect the exact number of height-influencing alleles inherited from the parents (13), parental height can be utilized to roughly estimate the adult height of children, the so-called genetic target height. Similar findings from a study in Korea showed non-catch-up SGA had a shorter father's height, mother's height, and mid-parental height in Koreans (22).

Our study had some limitations. First, it is a retrospective design. The lack of long-term follow-up data restricted the achievement of more accurate conclusions. Second, we did not collect and analyze nutrition data of this study cohort from birth to 2 years of age. While long-term lack of nutrition after birth might cause short stature in SGA children. Third, the sample size for investigation should be further expanded. Especially, the absence of adequate case number of giant placental chorioangioma for identifying the risk factor in short stature children born SGA at full-term was another weakness of this study. Besides, because of incomplete clinical data, we did not analyze IGF values, which was an important clinical detection for short stature.

In conclusion, the risk factors of full-term SGA infants developing short stature at age of 2 years included decreased placental perfusion fraction *f* p of IVIM parameters, chromosome abnormalities, and short parental height.

## Data Availability Statement

The original contributions presented in the study are included in the article/supplementary material, further inquiries can be directed to the corresponding author.

## Ethics Statement

The studies involving human participants were reviewed and approved by Ethical Committee of the First Affiliated Hospital of Nanjing Medical University. Written informed consent to participate in this study was provided by the participants' legal guardian/next of kin.

## Author Contributions

CL, LL, TC, X-HZ, X-QC, and S-DC conceptualized and designed the study, collected, analyzed, interpreted data, and performed the literature search. M-HP provided pathological data. All authors approved the final version to be submitted.

## Funding

This work was supported by the National Natural Science Foundation of China (81770162, 81170487).

## Conflict of Interest

The authors declare that the research was conducted in the absence of any commercial or financial relationships that could be construed as a potential conflict of interest.

## Publisher's Note

All claims expressed in this article are solely those of the authors and do not necessarily represent those of their affiliated organizations, or those of the publisher, the editors and the reviewers. Any product that may be evaluated in this article, or claim that may be made by its manufacturer, is not guaranteed or endorsed by the publisher.

## References

[1] Yadav S Rustogi D. Small for gestational age: growth and puberty issues. Indian Pediatr. (2015) 52:135–40. 10.1007/s13312-015-0588-z 25691182

[2] Finken MJJ van der Steen M Smeets CCJ Walenkamp MJE de Bruin C Hokken-Koelega ACS. Children born small for gestational age: differential diagnosis, molecular genetic evaluation, and implications. Endocr Rev. (2018) 39:851–94. 10.1210/er.2018-00083 29982551

[3] Campisi SC Carbone SE Zlotkin S. Catch-Up growth in full-term small for gestational age infants: a systematic review. Adv Nutr. (2019) 10:104–11. 10.1093/advances/nmy091 30649167 PMC6370265

[4] Huang L Yang S Yang F Xiong F. A prospective study about physical growth of children from birth to 2 years old born full-term small-for-gestational-age. J Paediatr Child Health. (2019) 5:199–204. 10.1111/jpc.14162 30066971

[5] Stalman SE Solanky N Ishida M Alemán-Charlet C Abu-Amero S Alders M. Genetic analyses in small-for-gestational-age newborns. J Clin Endocrinol Metab. (2018) 103:917–25. 10.1210/jc.2017-01843 29342293

[6] O'Callaghan JL Clifton VL Prentis P Ewing A Miller YD Pelzer ES. Modulation of placental gene expression in small-for-gestational-age infants. Genes (Basel). (2020) 11:80. 10.3390/genes11010080 31936801 PMC7017208

[7] Ma Y Pei Y Yin C Jiang Y Wang J Li X. Subchromosomal anomalies in small for gestational-age fetuses and newborns. Arch Gynecol Obstet. (2019) 300:633–39. 10.1007/s00404-019-05235-4 31273521

[8] Siauve N Hayot PH Deloison B Chalouhi GE Alison M Balvay D. Assessment of human placental perfusion by intravoxel incoherent motion MR imaging. J Matern Fetal Neonatal Med. (2019) 32:293–300. 10.1080/14767058.2017.1378334 28974131

[9] Chen T Zhao M Song J Mu X Jiang Y Zhou X. The effect of maternal hyperoxygenation on placental perfusion in normal and fetal growth restricted pregnancies using intravoxel incoherent motion. Placenta. (2019) 88:28–35. 10.1016/j.placenta.2019.08.078 31606612

[10] Peng R Yang J Xie HN Lin MF Zheng J. Chromosomal and subchromosomal anomalies associated to small for gestational age fetuses with no additional structural anomalies. Prenat Diagn. (2017) 37:1219–24. 10.1002/pd.5169 29025195

[11] Borrell A Grande M Meler E Sabrià J Mazarico E Muñoz A. Genomic microarray in fetuses with early growth restriction: a multicenter study. Fetal Diagn Ther. (2017) 42:174–80. 10.1159/000452217 27802431

[12] Arai S Sato Y Muramatsu H Yamamoto H Aoki F Okai Y. Risk factors for absence of catch-up growth in small for gestational age very low-birthweight infants. Pediatr Int. (2019) 61:889–94. 10.1111/ped.13939 31515924

[13] Lu T Forgetta V Wu H Perry JRB Ong KK Greenwood CMT. A polygenic risk score to predict future adult short stature among children. J Clin Endocrinol Metab. (2021) 106:1918–28. 10.1210/clinem/dgab215 33788949 PMC8266463

[14] Yu H Yuan M Wang L Li X Jiang M. Correlation between parturients' uterine artery blood flow spectra in the first and second trimesters of pregnancy and fetal growth restriction. J Healthc Eng. (2021) 2021:2129201. 10.1155/2021/2129201 34950439 PMC8692016

[15] Audette MC Kingdom JC. Screening for fetal growth restriction and placental insufficiency. Semin Fetal Neonatal Med. (2018) 23:119–25. 10.1016/j.siny.2017.11.004 29221766

[16] Zanardini C Papageorghiou A. Thilaganathan, B. Giant placental chorioangioma: natural history and pregnancy outcome. Ultrasound Obstet Gynecol. (2010) 35:332–6. 10.1002/uog.7451 19859897

[17] Buca D Iacovella C Khalil A Rizzo G Sirotkina M Makatsariya A. Perinatal outcome of pregnancies complicated by placental chorioangioma: systematic review and meta-analysis. Ultrasound Obstet Gynecol. (2020) 55:441–9. 10.1002/uog.20304 31034661

[18] de Wit MC Srebniak MI Joosten M Govaerts LC Kornelisse RF Papatsonis DN. Prenatal and postnatal findings in small-for-gestational-age fetuses without structural ultrasound anomalies at 18-24 weeks. Ultrasound Obstet Gynecol. (2017) 49:342–48. 10.1002/uog.15949 27102944

[19] Ester WA van Duyvenvoorde HA de Wit CC Broekman AJ Ruivenkamp CA Govaerts LC. Two short children born small for gestational age with insulin-like growth factor 1 receptor haploinsufficiency illustrate the heterogeneity of its phenotype. J Clin Endocrinol Metab. (2009) 94:4717–27. 10.1210/jc.2008-1502 19864454

[20] Butler MG Miller JL Forster JL. Prader-Willi syndrome - clinical genetics, diagnosis and treatment approaches: an update. Curr Pediatr Rev. (2019) 15:207–44. 10.2174/1573396315666190716120925 31333129 PMC7040524

[21] Angulo M Abuzzahab MJ Pietropoli A Ostrow V Kelepouris N Tauber M. Outcomes in children treated with growth hormone for Prader-Willi syndrome: data from the ANSWER Program and NordiNet international outcome study. Int J Pediatr Endocrinol. (2020) 2020:20. 10.1186/s13633-020-00090-6 33292530 PMC7653711

[22] Kim JH Kim DH Lim JS. Growth status of children and adolescents born small for gestational age at full term in Korea: data from the KNHANES-V. J Pediatr Endocrinol Metab. (2020) 33:743–50. 10.1515/jpem-2019-0471 32447332

